# Use of information and communication technologies to support effective work practice innovation in the health sector: a multi-site study

**DOI:** 10.1186/1472-6963-9-201

**Published:** 2009-11-08

**Authors:** Johanna I Westbrook, Jeffrey Braithwaite, Kathryn Gibson, Richard Paoloni, Joanne Callen, Andrew Georgiou, Nerida Creswick, Louise Robertson

**Affiliations:** 1 Health Informatics Research & Evaluation Unit, Faculty of Health Sciences, The University of Sydney, 75 East St, Lidcombe, NSW 1825, Australia; 2 Centre for Clinical Governance Research, Australian Institute of Health Innovation, Faculty of Medicine, University of New South Wales, 10 Arthur St, Kensington, NSW 2052, Australia; 3 Rheumatology Department, Liverpool Hospital, Locked Bag 7103, Liverpool BC, NSW 1871, Australia; 4 Emergency Department, Concord Hospital, Hospital Rd, Concord, NSW 2139, Australia; 5 Information Services Department, Royal Prince Alfred Hospital, Camperdown, NSW 2040, Australia

## Abstract

**Background:**

Widespread adoption of information and communication technologies (ICT) is a key strategy to meet the challenges facing health systems internationally of increasing demands, rising costs, limited resources and workforce shortages. Despite the rapid increase in ICT investment, uptake and acceptance has been slow and the benefits fewer than expected. Absent from the research literature has been a multi-site investigation of how ICT can support and drive innovative work practice. This Australian-based project will assess the factors that allow health service organisations to harness ICT, and the extent to which such systems drive the creation of new sustainable models of service delivery which increase capacity and provide rapid, safe, effective, affordable and sustainable health care.

**Design:**

A multi-method approach will measure current ICT impact on workforce practices and develop and test new models of ICT use which support innovations in work practice. The research will focus on three large-scale commercial ICT systems being adopted in Australia and other countries: computerised ordering systems, ambulatory electronic medical record systems, and emergency medicine information systems. We will measure and analyse each system's role in supporting five key attributes of work practice innovation: changes in professionals' roles and responsibilities; integration of best practice into routine care; safe care practices; team-based care delivery; and active involvement of consumers in care.

**Discussion:**

A socio-technical approach to the use of ICT will be adopted to examine and interpret the workforce and organisational complexities of the health sector. The project will also focus on ICT as a potentially *disruptive innovation *that challenges the way in which health care is delivered and consequently leads some health professionals to view it as a threat to traditional roles and responsibilities and a risk to existing models of care delivery. Such views have stifled debate as well as wider explorations of ICT's potential benefits, yet firm evidence of the effects of role changes on health service outcomes is limited. This project will provide important evidence about the role of ICT in supporting new models of care delivery across multiple healthcare organizations and about the ways in which innovative work practice change is diffused.

## Background

### The promise

Health systems globally are facing increasing demands for highly sophisticated services, yet they have limited resources and current and projected shortages of health professionals[1]. In OECD[2] countries the costs of healthcare delivery are rising, fuelled by ageing populations, more complex care and new medical technologies. In Australia, for example, health is already one of the most expensive sectors of the economy, at 9.3% of GDP;[3,4] and by 2045 this allocation is predicted to rise to at least 16%[5] One of the single most important challenges for health systems, then, is to establish new models of service delivery which increase capacity and provide rapid, safe, effective and affordable health care,[6,7] and do so sustainably, within health workforce and resource constraints. A key strategy being advanced to meet this challenge is increased use of information and communication technologies (ICT)[6].

Global expenditure on ICT across all sectors exceeds $US3.5 trillion[8] (2007 estimates) and is being driven by the desire for improvements in productivity, work practices and service outcomes [9-12]. Seeking the productivity gains and improved service outcomes evident in other industries, developed health systems such as those of the US, Canada and Australia are increasingly investing in ICT [13-15].

### The reality

Studies have shown that the use of ICT in the health sector is capable of increasing efficiency, reducing errors, supporting more team-based care, improving integration of best practice into routine care, enabling consumers to engage more actively in their care, and producing more efficient services through changes in professional roles and responsibilities[16,17]. However, this has been demonstrated only in exemplar organisations and isolated projects [16-18]. Evidence of large-scale changes in work practices, supported by ICT use, is lacking[16]. Information to date suggests that despite the rapid increase in ICT investment, uptake has been slow and the benefits fewer than expected[2,19]. Further, cases have emerged in which ICT has produced unexpected and negative effects in efficiency and safety [20-22].

Approaches to ICT implementation used in other industries have had limited success in the health sector. This is due in part to the sector's unique organisational and workforce characteristics. Healthcare organisations are complex[23]. The major professional groups have high levels of autonomy[24,25], are tribal in their behaviours[26] and operate in hierarchical structures[26]. Work is highly specialised and work processes non-linear[27,28]. Yet safe and effective work is dependent upon horizontal work co-ordination, particularly strong collaborations between professional groups; thus effective inter-professional and organisational communication is vital[29]. Furthermore, unlike in some industries, ICT in health seems to lead to an *increase *in the complexity and intellectual content of work, rather than to the simplification or removal of complex tasks[30,31]. The business process reengineering[32] approaches to work practice change that have been prominent in health ICT projects are, in contrast, usually based on top-down linear workflow models[30] and are often inadequate for dealing with the complex collaborative nature of medical work. The limitation of these traditional approaches to ICT work practice reform is evidenced in the large number of reported failures of large health IT projects [33-35]. Constant changes in the systems used cause additional problems. For example, a survey of over 800 participants at an annual electronic medical record (EMR) trade fair in the US in 2007 found that 19% of respondents reported that they had or were in the process of de-installing an EMR system[36].

These failures have led to a search for approaches that place greater emphasis on the interconnectedness between the social (people, values, norms, culture) and technical (tools, hardware, equipment, processes) aspects of organisations[37,38]. For example, Computer-Supported Cooperative Work (CSCW) studies have investigated the ways in which individuals and teams interact with technologies such as flight simulators[39,40]. But one problem with human-technology interaction studies is that they are based largely on a theory of command and control[41]. For example, in the cockpit a small team conducts a sequence of tasks in accordance with explicit rules and regulations. This workplace is clearly defined and relatively isolated. Studies focussed on such bounded organisational structures are of questionable relevance to more intricate and fragmented work settings. The health sector, with its many professional subgroups, complex work processes and power structures, represents a much more fluid and dynamic context with fewer formalised control mechanisms[42].

### Gaps in knowledge

Although it is recognised that the benefits of ICT will not be realised without considerable changes in work processes and structures,[19] there is a critical absence of research-based, empirically-tested models for achieving this on a large-scale in the health sector[43,44]. Instead, single site, short-term, primarily descriptive studies have dominated research in the field internationally. Also, researchers have focussed almost exclusively on studying organisations that have developed their own ICT systems. In a systematic review of the impact of ICT use in health, nearly 25% of studies were conducted in one of four US medical centres, all with home-grown systems[16]. In only 9% of 257 studies were commercial systems examined. Yet in Australia, as in other countries, it is commercial systems that are implemented by the vast majority of organisations. Most of these systems are developed in the United States and thus are designed for health delivery models that may not hold in other health jurisdictions. As such, they create particular challenges for predominantly publicly-funded health systems and may create barriers to their use in supporting effective work practice change[45,46]. In summary, there is poor understanding of why some organisations are able to achieve significant work practice change, yet others using the same ICT systems are not[46]. This raises questions about which factors enable or inhibit ICT-supported work innovation. In previous studies we have shown that characteristics of team[47] and organisational[48] cultures are associated with effective ICT use, but there are likely to be other significant factors.

Thus the evidence of how ICT can support and drive innovative work practice change is generally weak and comes largely from non-transferable case studies of single organisations[16,21,49]. Large-scale multi-site studies are now needed. This is crucial not only to achieve the productivity improvements required, but to create safer health systems[50]. One in ten patients is harmed as a result of care received, and studies have shown overwhelmingly that poor communication and lack of teamwork are major causes[51], Runciman, 2007 #1852] ICT is central to improving communication and teamwork to deliver a safer health system.

The aim of this research is to conduct a large-scale, multi-site study to measure current ICT impact on workforce practices. It will also develop and test new models of ICT use which support innovations in work practice.

## Methods/Design

### Setting

The research will be undertaken within the Sydney South West Area Health Service (SSWAHS) which provides publicly-funded health care services for a population of 1.4 million people in central and south western Sydney. The population represents the most ethnically diverse Area Health Service in Australia. SSWAHS operates and manages 17 health care organisations with a workforce in excess of 17,000 people.

### Interventions

**Figure 1:**
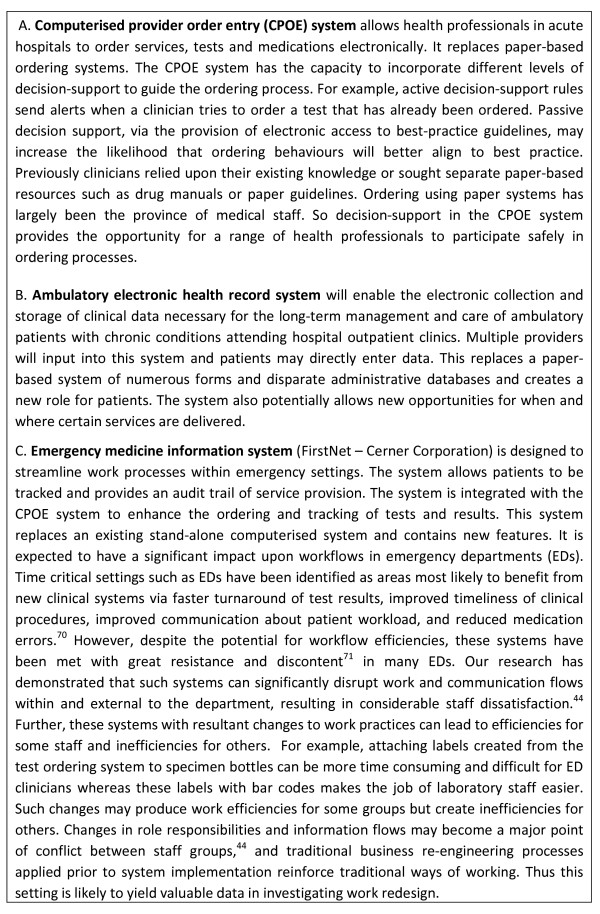
**The ICT Interventions**.

The information and communication technologies which will be the focus of this research are: computerised ordering systems; ambulatory electronic medical record systems; and emergency medicine information systems. All three ICT interventions present significant opportunities for work innovation. Figure 1 provides details of these systems.

### Study objectives

**Figure 2:**
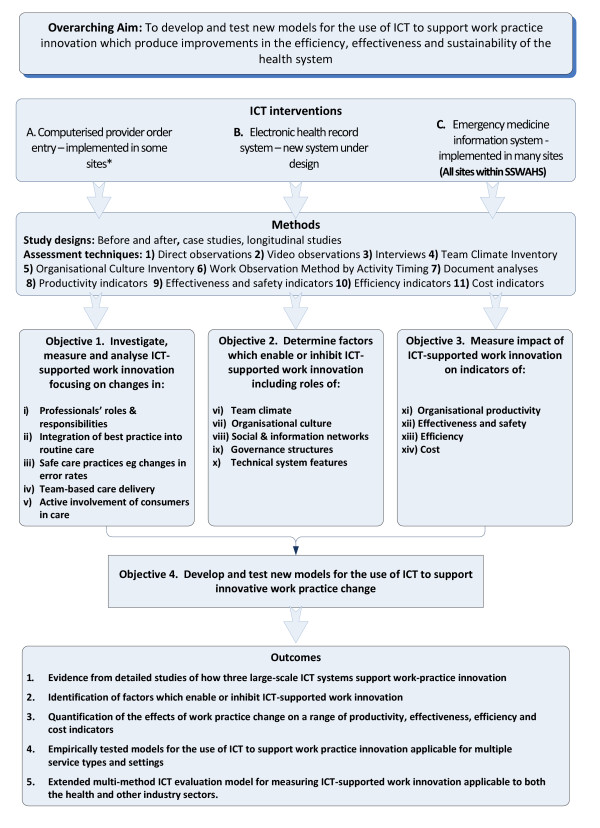
**Conceptual Research Design and Methods**.

We will focus on the role of ICT to support five attributes of work practice innovation (see Figure 2, i-v):

**Objective 1 **To investigate, measure and analyse the role of ICT in supporting work practice innovation

**Objective 2 **To determine the factors that enable or inhibit ICT-supported work innovation. Informed by the literature and our past research[38,46] we have identified five areas which will be the target of investigation here (Figure 2, vi-x).

**Objective 3 **To quantify the impact of ICT-supported work innovation on organisational productivity, effectiveness, efficiency and cost (Figure 2, xi-xiv).

**Objective 4 **To apply the findings from objectives 1-3 to develop and test new models for the use of ICT to support innovative work practice change. These models may include, for example, methods for identifying opportunities for role responsibility changes using ICT and provision of tools for testing the impact of changes on service outcomes. We will test the effectiveness of these models in different settings using methods summarised in Figure 2.

The timetabling of interventions will allow for controlled before and after studies, case studies and longitudinal studies. Importantly, the interventions cover the wide breadth of services provided, from emergency to acute inpatient and outpatient care. We will be able to compare different organisations while keeping the ICT system and overall Area Health Service organisational factors constant. This will assist in isolating the effects of local team-based cultures on work innovation.

### Data collection methods

Direct observations of practice in multiple sites will be undertaken to identify work role changes and innovation. This will be accompanied by video observation and interviews[27,38,52,53]. These data will provide insights into changes in roles and responsibilities, team relationships, consumer involvement and factors identified by clinicians as supporting or preventing work innovation. The data will be combined with organisational document analyses, and non-participant observation of project steering and organisational committees. System functionality evaluations will be undertaken in conjunction with user feedback (via surveys, observations and interviews) about system performance, specifically in relation to integration of ICT with work practices. Observational studies and interviews will focus on identification of workaround procedures, as we have found[54,55] these are often introduced to accommodate systems which fail to integrate with work practices, or where practices have not been changed to take advantage of work process efficiencies which ICT offer.

We will apply social network analyses, in which we have expertise,[56,57] to examine social and communication networks. Drawing upon our past results we will focus on work task areas identified as opportunities for work innovation. The social network analyses will examine the extent to which participants across the enrolled organisations are linked and how discussions and ideas about work practice change are communicated between and across discipline areas, departments and services. The social network analyses will also investigate and provide a measure of team-based care. This will be quantified further using the Team Climate Inventory (TCI), a tool to gauge the extent of team cohesiveness and innovation. We have shown that teams with high TCI scores report more innovative use of ICT[47]. The Organisational Culture Inventory (OCI)[48], which we have demonstrated[58] is able to discriminate between cultures in health organisations, will also be administered.

Quantitative changes in work patterns of clinicians will be calculated using our Work Observation Method by Activity Timing (WOMBAT), which we have developed and tested for reliability and validity [59-61]. This method uses direct structured observations of individuals. The observer records information about what, with whom and how each task is undertaken, as well as interruptions and multi-tasking, using a personal digital assistant (PDA). The PDA automatically time stamps tasks and thus allows quantification of work patterns.

We will measure the effects of ICT-supported work innovation using a range of indicators including changes in:

1. organisational productivity measures such as number of patients treated and tests processed, lengths of patients' stays in hospitals, emergency department and outpatient visit length, and staffing levels and mix;

2. effectiveness and safety indicators such as changes in rates of medication error and unnecessary duplicate test orders; and

3. efficiency indicators such as turnaround time of test results, and staff time consumed by specific categories of work.

These data will be costed and combined with system implementation and maintenance costs to measure cost-effectiveness of ICT-supported work innovations. We will use both local financial data and costing data produced in previous studies[62].

Findings will be tested as they emerge by soliciting feedback from stakeholder groups. Staff at the participating sites will be recruited as active contributors to the process of interpreting data and informing the research conclusions.

#### Exemplar sub-studies

##### Ambulatory electronic medical record

As part of the rheumatology ambulatory electronic medical record (eMR), an electronic toxic drug monitoring system (eTDMS) has been developed to assist clinicians in monitoring rheumatology patients who are placed on toxic drugs, known as Disease Modifying Anti-rheumatic Drugs (DMARDs). The aims of this sub-study are to evaluate the effectiveness of the eTDMS in terms of: appropriate drug monitoring; time taken by nursing staff to monitor patients and the impact of the eTDMS on clinicians' work processes. Applying a before and after study design the first stage of this study will determine whether toxic drug monitoring has improved using the eMR. The sample size for this sub-study, based on power of 80% to detect a difference between pre and post intervention sample proportions at p = 0.05 is 60 patients in each study period. Work process changes will be identified using work process maps, interviews with clinicians, and time and motion work measurement studies of nurses in the clinic. The intervention will then be trialled at a second rheumatology clinic in another hospital and results of that trial compared.

##### Emergency medicine clinical information systems

A cross-sectional qualitative study, utilising interviews, focus groups, observation and video ethnography, will be conducted in the Emergency Departments (ED) of eight hospitals. Each technique will focus on eliciting information about clinicians' views and practices in relation to ICT systems used, how they have changed everyday work practices, specific effects on communication, professional roles and patient care outcomes. Interviews and focus groups will involve a sample of approximately 100 clinical staff. Further, a social network analysis to measure the ways in which senior emergency clinicians are connected across the eight hospitals will be conducted and the extent to which social networks influence the diffusion of new work practices with ICT assessed. This will involve a sample of approximately 70 senior emergency clinicians from across the eight hospitals.

Ethics approval for the overall study was granted by the Sydney South West Area Health Service Human Research Ethics Committee Multi-centre project No.09/CRGH/53, CH62/6/2009-046. This includes provision of informed consent where required by participants.

## Discussion

We will apply a socio-technical approach to the use of ICT in health. This provides a powerful theoretical paradigm by which to examine and interpret the workforce and organisational complexities of the health sector, and it will overcome some of the limitations of previous approaches. We will extend this conceptual work in the proposed project by exploring the role of ICT as a potential *disruptive innovation*[63]. This term refers to technological innovations that challenge and often eventually overturn the status quo. Such technologies are often embraced by consumers while being viewed as threatening by industry incumbents. For example, commentators external to the health sector have argued that such technologies are needed in health in order to change traditional patterns of work and *"enable less expensive professionals to do progressively more sophisticated things in less expensive settings" *(p15)[64]. Professional bodies, on the other hand, have voiced concerns about possible role changes[1]. The limited evidence as to the effects of role changes on service outcomes continues to stifle discussion as well as wider explorations of how ICT can be used to provide more efficient health services. Our research will provide important evidence to inform this debate and provide the evidence-base for relevant health policy.

A further planned outcome of this research is evidence of how innovative work practice change is diffused. Existing research evidence demonstrates that the adoption and diffusion of new ideas and innovation is related to the patterns of communication within and across organisations[65]. Studies outside health[66,67] have shown that those organisations with the highest rates of innovation have cultures which encourage collaborations, the free flow of information, and groups who work together on new ideas. Drawing upon uncertainty reduction theory[68], which argues that individuals communicate in order to reduce uncertainty and that this process ties people together and promotes further interactions, Albrecht et al[69] studied three large organisations and found that staff were most likely to report the embracing of new ideas if discussion about work and social matters also occurred. The proposed research, unlike previous single-site studies, will have the capacity to examine the ways in which innovative work practices and ideas are distributed within and across communities and organizations within an area health service.

## Conclusion

This Australian-based project will address a highly significant problem for health sectors internationally, namely how to harness ICT to create new and more effective models of service delivery which increase capacity and provide rapid, safe, effective and affordable health care, but which do so sustainably, within health workforce and resource constraints. The project will measure current ICT impact on workforce practices and develop and test new implementation models. This research will contribute to the health informatics knowledge-base by examining the ways in which ICT can support work innovation to achieve new models of health service delivery which produce measurable improvements. In addition, it will measure how innovative practices are disseminated.

## List of abbreviations used

ICT: Information and communication technologies; SSWAHS: Sydney South West Area Health Service; EMR: electronic medical record.

## Competing interests

The authors declare that they have no competing interests.

## Authors' contributions

JW and JB conceptualized the project, led the design of the study and wrote the protocol with significant input from KG, RP, AG, JC, NC and LR. All authors contributed to reviewing the protocol and approved the final version of the manuscript.

## Pre-publication history

The pre-publication history for this paper can be accessed here:


